# Caregiver burden among caregivers of children with autism spectrum disorder

**DOI:** 10.4102/sajpsychiatry.v29i0.2079

**Published:** 2023-10-20

**Authors:** Karli van Niekerk, Venera Stancheva, Cornelia Smith

**Affiliations:** 1 Department of Psychiatry, Faculty of Health Sciences, University of Witwatersrand, Johannesburg, South Africa

**Keywords:** autism spectrum disorders, caregiver burden, children, Zarit Burden Interview, South Africa

## Abstract

**Background:**

Autism spectrum disorder (ASD) is a neurodevelopmental disorder with deficits in social communication and interaction, restricted and repetitive patterns of behaviour, interests and activities. Autism spectrum disorder is associated with multiple comorbidities. As a result, caregivers of children with ASD experience increased levels of burden and poor quality of life. However, there is a paucity of information on the burden.

**Aim:**

The study aimed to describe the sociodemographic profiles and determine the extent of the burden experienced by caregivers of children and adolescents with ASD.

**Setting:**

The Child, Adolescent and Family Unit (CAFU) outpatient services at Charlotte Maxeke Johannesburg Academic Hospital (CMJAH).

**Methods:**

A quantitative, descriptive, cross-sectional study was done. Two self-administered questionnaires were used: a sociodemographic questionnaire and the 12-item Zarit Burden Interview questionnaire.

**Results:**

The questionnaires were completed by 77 caregivers, of which the majority were female (*n* = 56 or 72.3%), mothers to children with ASD (*n* = 49 or 64.3%) and identified as Christian (*p* < 0.001). Most had completed secondary school or had a tertiary education (*p* = 0.003) and were employed (*p* < 0.001). Among the caregivers, 41.6% experienced mild to moderate burden, 33.8% experienced high burden and only 24.9% reported no to mild burden.

**Conclusion:**

Caregivers of children and adolescents with a diagnosis of ASD are mostly mothers and experience mild to moderate levels of caregiver burden, suggesting the need for improved screening and psychosocial support programmes.

**Contribution:**

This study highlights the burden experienced by primary caregivers of children with ASD and is one of the few comprehensive studies on this issue within the context of South Africa.

## Introduction

Autism spectrum disorder (ASD) is a complex, heterogeneous, multifaceted neurodevelopmental disorder with onset during early childhood.^1,2^ It is characterised by significant and persistent deficits in social communication and social interaction, as well as restricted, repetitive patterns of behaviour, interests and activities.^3^

The Diagnostic and Statistical Manual of Mental Disorders fifth edition (DSM-5^TM^) divides the severity of ASD into three levels based on the degree of impairment in adaptive and cognitive functioning.^3^ Individuals with level 1 severity require some support, while individuals with level 2 need substantial support and those with level 3 need very substantial support.^3^

The global prevalence of ASD is increasing because of a better understanding of the disorder and the expansion of the diagnostic criteria.^4^ Elsabbagh et al.^5^ estimated that the mean global prevalence translated into 1 child out of 160 having ASD, while Franz et al.^6^ reported the prevalence to be between 1% and 2% of the global population. In Africa, studies on the prevalence of child and adolescent mental disorders have been conducted on small samples and are, therefore, poorly representative of the disease burden.^5^ Although prevalence studies in South Africa are limited, a survey by Pillay et al.^7^ in the Western Cape showed that the prevalence of ASD increased by 76.03% from 2012 to 2016, with an annual increase of 15.18%.

There is a high rate of medical and psychiatric comorbidities associated with ASD. Medical disorders include refractory epilepsy, sleep disorders and gastrointestinal disorders, whereas psychiatric disorders include intellectual disability, attention-deficit hyperactive disorder, motor disorders, behavioural problems, anxiety disorders and mood disorders.^8,9,10,11,12,13,14,15,16,17,18^ In addition, the complexity of the disorder, associated comorbidities and lifelong nature contribute to the burden experienced by caregivers.^19^

There are no definite protocols for treating ASD and its associated comorbidities.^20^ Pharmacological interventions are limited to treating comorbidities and lessening symptom severity, but they are ineffective in addressing the core symptoms of ASD.^21^

A well-informed caregiver is an essential part of managing a child with ASD. A caregiver is defined by Kent et al.^22^ as the person responsible for looking after someone who cannot function independently and requires assistance with activities of daily living. Caregivers are required to meet the needs of the child with ASD, manage challenging behaviour and follow through with professional recommendations from healthcare providers.^23^

Research on caregiver burden began receiving attention in the late 1970s, and in recent years, more research has been generated on caregivers looking after patients with medical and psychiatric disorders.^24,25^ However, research in children and adolescents primarily focuses on medical conditions, with research on psychiatric disorders and caregiver burden needing to be improved.^26^

Previous research on caregivers has reported on the comprehensive challenges experienced by caregivers, including negative impacts on their physical and emotional well-being, difficulties in relationships with other family members, work challenges and financial constraints because of loss of income.^27^ Primary caregivers face multiple challenges and experience impairments in their quality of life.^23^

Research has shown that biological mothers are usually the primary caregivers of children with ASD.^28^ It has been concluded that they often experience higher levels of burden than mothers of typically developing children.^28^ Caregivers of children with ASD have reported elevated fatigue levels and increased physical health complaints compared to caregivers of neurotypical and intellectually disabled children.^29^

Special attention has been given to the incidence of mental disorders in caregivers of children with ASD. Karst et al.^30^ reported a higher incidence rate of depression and anxiety among caregivers of children with developmental disabilities, including ASD. Lai et al.^31^ recognised that caregivers reported more depressive symptoms and that anxiety symptoms tend to fluctuate throughout the child’s development. Sharpley et al.^32^ concluded that challenging behavioural problems are associated with a higher prevalence of depression and anxiety. Paruk and Ramdhial^33^ conducted a study in South Africa that determined mothers were mostly the primary caregivers of children with psychiatric disorders and that they experienced high levels of burden and depressive and anxiety symptoms.

Challenges associated with behaviour and the stigmatisation of children with ASD often result in social isolation and decreased quality of life for caregivers.^34^ However, a more recent study by Ten Hoopen et al.^28^ did not find a significant decrease in health-related quality of life in primary caregivers of children with ASD. This finding was attributed to the measuring tools used and the sample characteristics of their study population.

Parents of children diagnosed with ASD reported lower marital satisfaction levels.^35^ The divorce rates among these families also appear to be nearly double compared to families with typically developing children, with younger parents at higher risk.^36^

Caregivers often incur financial burdens. As a result, they are more likely to experience a loss of income and ultimately less access to supportive resources, including appropriate school placements, specialised care facilities and social-emotional support.^34^

Identifying the specific areas of burden experienced by caregivers of children with ASD can help develop tailored support structures to meet their unique needs. By providing adequate support, caregivers can better manage the challenges associated with caring for a child with ASD and improve their quality of life.

In the literature, caregiver burden is measured by well-defined tools, with the Zarit Burden Interview (ZBI) questionnaire being one of them.^37^ The ZBI has been used in multiple international research studies over a wide range of sociodemographic profiles.^38,39^ However, while South African researchers have used the ZBI questionnaire to determine caregiver burden among caregivers of orphaned children in rural South Africa, there has yet to be any known research on its use in the psychiatric caregiving population in South Africa.^40^

There is a paucity of research in South Africa measuring caregiver burden in child and adolescent psychiatry. Nonetheless, because of the unique challenges of caring for a child with ASD, it is essential to research this population to better understand the extent of the burden and develop appropriate support structures.

## Aim

The study aimed to describe the sociodemographic profiles and to determine the extent of caregiver burden among caregivers of children and adolescents diagnosed with ASD at the Charlotte Maxeke Johannesburg Academic Hospital (CMJAH) in South Africa.

## Research method and design

### Study design and setting

A quantitative, descriptive, cross-sectional study was conducted. The participants were asked to complete a self-administered sociodemographic questionnaire and the 12-item ZBI questionnaire.^24^

The sociodemographic questionnaire comprised questions on the following variables: age, gender, marital status, religion, the highest level of education, employment status, monthly income or disability grant and the relationship between the caregiver and the patient with ASD.

The ZBI questionnaire is a standardised questionnaire validated for research use in the caregiving population by Seng et al.^41^ Previous research has identified that the 12-item short-form version was suitable for screening for caregiver burden, demonstrated good psychometric properties and was validated in cross-sectional and longitudinal studies.^37^ The 12-item version consists of two domains, personal and role strain and is measured on a five-point Likert scale. The range of summed scores is between 0 and 48, with a total score of 0–10 representing no to mild burden, 11 to 20 representing mild to moderate burden and above 20 representing high burden.^42^

The study was conducted at the Child, Adolescent and Family Unit (CAFU) at CMJAH from October 2021 to February 2022. The unit provides outpatient services and is part of the Division of Child and Adolescent Psychiatry at the Department of Psychiatry, University of Witwatersrand Medical School.

### Study population

All consenting caregivers older than 18 years of age looking after a child or adolescent diagnosed with ASD attending the unit at CMJAH were included in the study.

Participants of all genders, ethnicities, religions and sociodemographic backgrounds were included. Participants unable to complete the questionnaires because of a language barrier were provided with a translator after obtaining consent. Caregivers declining to participate were excluded from the study and only participants who completed both questionnaires were included in the data analysis.

The sample size was determined by assessing z-scores of expected frequencies per question in the ZBI questionnaire. Statistical significance could be expected with a minimum sample of 70, assuming a large effect size.

### Data collection

The consenting caregivers completed the questionnaires in a private room. Where assistance with completing the questionnaires was required, the researcher or the allocated case manager aided. The complete questionnaires were placed in a sealed box.

### Data analysis

Categorical data scores were analysed using Pearson’s chi-square tests for sociodemographic variables and the multiple responses for each ZBI question. Continuous variables (e.g. age) were summarised as mean (SD). In addition, Likert plots were generated for various responses to the ZBI question. All tests were two-tailed probability values and statistical significance was accepted when α ≤ 0.05.

### Ethical considerations

Permission for this study was granted by the Human Research Ethics Committee (Medical) of the University of the Witwatersrand (no. M210841).

Written informed consent was obtained for every participant in the study. Each participant was allocated a study number and no identifying data was used.

A distress protocol with available resources and contact information was provided to all participants.

## Results

**FIGURE 1:**
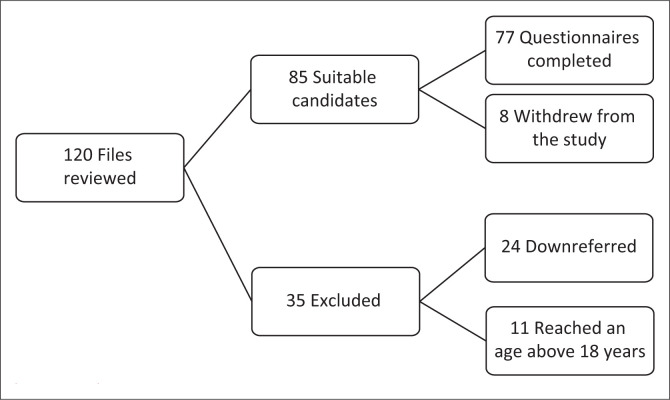
Flowchart outlining participant selection.

The clinical records of 120 patients with a primary diagnosis of ASD were reviewed (Figure 1). Thirty-five patients were excluded from the study, with 24 being down-referred to community clinics and 11 being transferred to adult psychiatric services after reaching the age of 18 years. The questionnaires were administered to 85 participants, of which 8 did not complete the questionnaires. Only 77 were included in the final data analysis.

### The sociodemographic characteristics

Table 1 presents the sociodemographic characteristics of the participants. Most caregivers were female (*n* = 56 or 72.73%), while only 21 (27.27%) were male. The mean age of the participants was 44.4 (SD ± 8.9) years. The females were younger with a mean age of 43.6 years and males were slightly older with a mean age of 47.2 years.

**TABLE 1:** Sociodemographic characteristics of the caregivers in a study population at the Charlotte Maxeke Johannesburg Academic Hospital.

Demographics	Count	%	*p*
**Age (*n* = 77)**			
Mean age	44.4	-	-
Mean age of males	47.2	-	-
Mean age of females	43.6	-	-
**Gender (*n* = 77)**	-	-	< 0.001
Male	21	27.27	-
Female	56	72.73	-
Other	0	0.00	-
**Marital status (*n* = 76)**	-	-	< 0.001
Single	34	44.74	-
Married	30	39.47	-
Divorced	4	5.26	-
Separated	2	2.63	-
Widowed	6	7.89	-
**Religion (*n* = 77)**	-	-	< 0.001
Christian	65	84.42	-
Muslim	3	3.90	-
Hindu	0	0.00	-
Jewish	2	2.60	-
Spiritual	3	3.90	-
African traditional beliefs	2	2.60	-
Other (specify)	2	2.60	-
**Highest level of education (*n* = 76)**	-	-	< 0.001
Primary school	4	5.26	-
Secondary school	37	48.68	-
Tertiary education	27	35.53	-
College	8	10.53	-
**Employment (*n* = 77)**	-	-	0.003
Full time employed	35	45.45	-
Part-time employed	12	15.58	-
Unemployed	30	38.96	-
**Income (*n* = 77)**	-	-	< 0.001
Disability grant	12	15.58	-
R0 – R3000	23	29.87	-
R3000 – R6000	13	16.88	-
R6000 – R9000	4	5.19	-
R9000 – R12 000	4	5.19	-
R12 000 – R15 000	3	3.90	-
Above R15 000	18	23.38	-
Other (specify)	0	0.00	-
**Relationship to the patient (*n* = 76)**	-	-	< 0.001
Mother	49	64.47	-
Father	16	21.05	-
Grandmother	6	7.89	-
Grandfather	1	1.32	-
Aunt	0	0.00	-
Uncle	0	0.00	-
Sibling	0	0.00	-
Foster parent	0	0.00	-
Other (specify)	4	5.26	-

Note: Sample sizes vary because of missing data.

In this sample, most caregivers were single (*n* = 34 or 44.74%), while more than one-third were married (39.47% or *n* = 30). Of the remaining caregivers, 7.89% (*n* = 6) were widowed, 5.26% (*n* = 4) were divorced and 2.63% (*n* = 2) were separated.

Most caregivers identified as Christian (84.42% or *n* = 65), while two (2.6%) identified as Jehovah’s Witnesses. A small percentage identified as Muslim or followed spiritual beliefs (3.9% or *n* = 3, respectively) and Jewish or African traditional beliefs (2.6% or *n* = 2 each).

Most caregivers (48.68% or *n* = 37) completed secondary education, while 46.06% (*n* = 35) completed tertiary education. On the other hand, very few caregivers (5.26%, *n* = 4) did not study further than primary school.

Out of all the caregivers, 61.03% (*n* = 47) were employed. Of those employed, 45.45% (*n* = 35) worked full time and 15.58% (*n* = 12) worked part time. Meanwhile, over one-third (38.9% or *n* = 30) were unemployed.

Approximately one-third of caregivers (29.87% or *n* = 23) received a monthly income of R3000 or less, with 15.58% (*n* = 12) solely relying on a child disability grant. An income between R6000 to R15 000 was reported by 31.16% (*n* = 24) and only 23.38% (*n* = 18) received a monthly income greater than R15 000.

Mothers comprised the majority of caregivers (64.47% or *n* = 49) for children and adolescents with ASD, while fathers accounted for only 21.05% (*n* = 16) of primary caregivers. In seven cases (9.21%), grandparents were identified as primary caregivers. Only 5.26% (*n* = 4) of the caregivers were not biological relatives of the children or adolescents, with 3 being formally employed full-time help and one being a stepfather to the child with ASD. Siblings, aunts, uncles and foster parents were not identified as caregivers in this sample population.

### The Zarit Burden Interview characteristics

**FIGURE 2:**
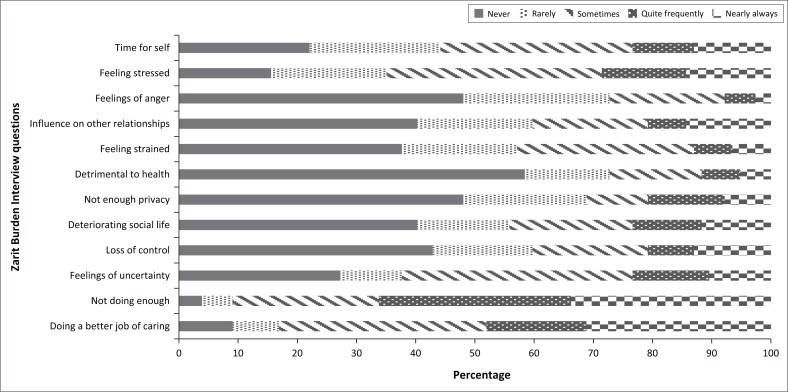
Likert plots of the responses to a Zarit Burden Interview questionnaire by caregivers of children with autism spectrum disorder at the Charlotte Maxeke Johannesburg Academic Hospital.

The findings are summarised in Table 2 and Figure 2.

**TABLE 2:** Zarit Burden Interview questionnaire.

Question: How often do you feel …	Never		Rarely		Sometimes		Quite frequently		Nearly always	
	*n*	%	*n*	%	*n*	%	*n*	%	*n*	%
… that because of the time you spend with your relative that you don’t have enough time for yourself?	17	22.08	17	22.08	25	32.47	8	10.9	10	12.99
… stressed between caring for your relative and trying to meet other responsibilities (work or family)?	12	15.58	15	19.48	28	36.36	11	14.29	11	14.29
… angry when you are around your relative?	37	48.05	19	24.68	15	19.48	4	5.19	2	2.60
… that your relative currently affects your relationship with family members or friends negatively?	31	40.26	15	19.48	15	19.48	5	6.49	11	14.29
… strained when you are around your relative?	29	37.66	15	19.48	23	29.87	5	6.49	5	6.49
… that your health has suffered because of your involvement with your relative?	45	58.44	11	14.29	12	15.58	5	6.49	4	5.19
… that you don’t have as much privacy as you would like because of your relative?	37	48.05	16	20.78	8	10.39	10	12.99	6	7.79
… that your social life has suffered because you are caring for your relative?	31	40.26	12	15.58	16	20.78	9	11.69	9	11.69
… that you have lost control of your life since your relative’s illness?	33	42.86	13	16.88	15	19.48	6	7.79	10	12.99
… uncertain about what to do about your relative?	21	27.27	8	10.39	30	38.96	10	12.99	8	10.39
… you should be doing more for your relative?	3	3.90	4	5.19	19	24.68	25	32.47	26	33.77
… you could do a better job in caring for your relative?	7	9.09	6	7.79	27	35.06	13	16.88	24	31.17

Of the caregivers in this sample, 25 (32.47%) reported feeling that they sometimes did not have enough time for themselves because of their caregiving responsibilities. However, only 10 (12.99%) reported experiencing these feelings nearly always.

Of all the caregivers, 36.36% (*n* = 28) reported experiencing frequent stress while caring for a child with ASD and trying to meet the demands of work and family responsibilities. Among the remaining caregivers, 14.29% (*n* = 11) selected ‘quite frequently’ and ‘nearly always’ as their stress levels.

Feelings of anger towards the child with ASD were uncommon, with 48.05% (*n* = 37) reporting ‘never’ feeling this way. In addition, 40.26% (*n* = 31) felt that caring for the child or adolescent did not negatively affect their other relationships. Only five (6.49%) participants reported feeling ‘quite frequently’ or ‘nearly always’ strained by their caregiving roles.

Most caregivers (*n* = 45 or 58.44%) did not report a decline in their physical health. Additionally, 37 participants (48.05%) did not feel that their privacy had been affected by caring for a child with ASD. Only 9 participants (11.69%) reported a significant negative impact on their social lives because of their caregiving role, while 31 (40.26%) reported ‘never’ feeling this way.

Although most participants (*n* = 48 or 62.34%) experienced uncertainty about their role as a caregiver and what to do for the child with ASD under their care, the majority (*n* = 33 or 42.86%) did not feel they had lost control of their own lives.

The study found that caregivers felt a strong sense of responsibility to do more for the child with ASD and to improve their caregiving performance. These findings were statistically significant, with a *p*-value of less than < 0.001.

### Burden prevalence

**FIGURE 3:**
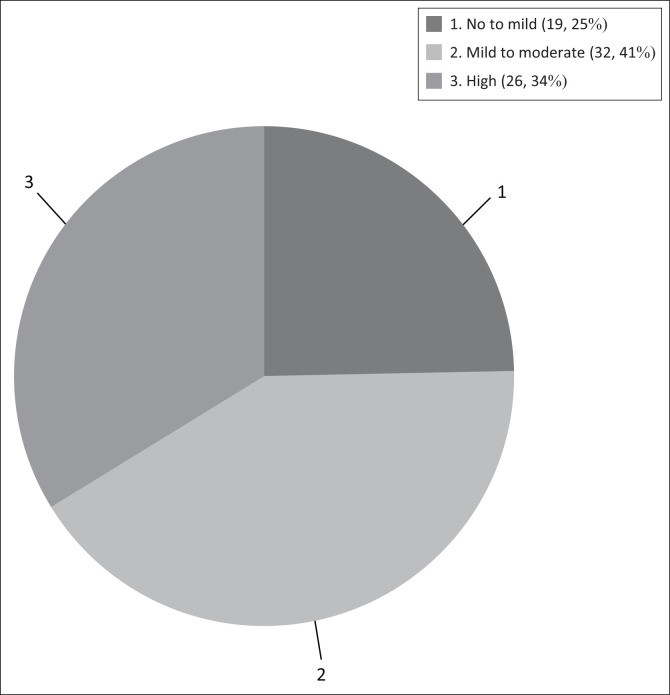
Burden prevalence.

The burden prevalence results are presented in Figure 3, with each participant receiving an individual score. The majority of participants (*n* = 32 or 41.56%) experienced mild to moderate burden, while 26 (33.77%) reported high levels of burden. Less than one-quarter of participants (*n* = 19 or 24.68%) experienced no to mild burden. It is worth noting that the distribution of the scores was not found to be significantly different from chance (*p* = 0.19).

Of the male participants, 38.09% (*n* = 8) experienced no to mild burden, 47.62% (*n* = 10) experienced mild to moderate burden and 14.29% (*n* = 3) experienced high levels of burden. The majority of female participants also experienced mild to moderate burden (*n* = 27 or 48.21%). Only 10.71% (*n* = 6) of females experienced no to mild burden and 41.07% (*n* = 23) reported high levels of burden.

## Discussion

Autism spectrum disorder is a complex neurodevelopmental disorder associated with challenging behaviour.^1^ Therefore, taking care of a child with ASD can be strenuous and caregivers often face multiple difficulties that subject them to higher stress levels.

### Sociodemographic characteristics

Different sociodemographic characteristics can contribute to the level of burden that caregivers experience.

Multiple research studies in Europe and Africa revealed that mothers are generally the primary caregivers of their children with ASD.^33,43^ The current study supported previous findings, with 72.7% of the caregivers being female, of which 64.5% were mothers of the children with ASD. An additional finding was that one-fifth of caregivers were fathers to the children with ASD. However, there is a paucity of data on single fathers caring for children with ASD. It has been identified that the roles and experiences of fathers as primary caregivers are often overlooked.^44^

A review article by Lai et al.^31^ concluded that caregivers of children with ASD mostly fall within the middle-aged group. The current study also showed that most caregivers were middle-aged, with female caregivers slightly younger than males.

A South African study done in 2017 reported that most children with ASD grew up in a two-parent household.^45^ However, our study found that most caregivers were single (never married, divorced, separated or widowed), with only 39.5% being married. This finding could reflect the general South African household, where 42% of children are raised by their mothers only.^46^

A multinational study done in the United Kingdom, the United States, Canada and Australia looked at the influence of spirituality and religion on caregivers of ASD. The study identified that most caregivers (61%) were of the Christian religion.^47^ The current research supported this finding. However, the conclusion that most caregivers were Christian is in keeping with the religious affiliation of the general population in South Africa and might not be a significant study finding.^48^

Past research showed that most caregivers have at least completed secondary education.^39,49^ The current study showed similar results, with most caregivers having an education level of high school and above. A higher level of education can contribute to caregivers’ acquired knowledge of their child’s condition and the availability of supportive resources leading to better management and quality of life for the child. This could be explained by the assumption that educated caregivers are more likely to be employed and have access to specialised care.

Previous publications showed that caregivers of children with ASD were often unemployed.^39,49^ However, a Green^50^ study identified that employment could be a protective factor when caring for a child with ASD. In this study, most participants were either full-time or part-time employed. However, over one-third of the participants were unemployed. This finding could reflect the current economic climate in South Africa, where there is a higher unemployment rate compared to other countries.^51^

Research in South Africa identified that the costs of special education, medication, therapeutic interventions and special dietary requirements for children with ASD are high.^52^ Twelve caregivers relied exclusively on a child disability grant and 23 received between no income and R3000 per month. However, research done by Geldenhuys concluded that the current amount received from a monthly disability grant is insufficient to meet all the child’s demands.^52^ This can put additional strain on caregivers.

### Zarit Burden Interview questionnaire characteristics

This study used the ZBI questionnaire to determine the extent of the caregiver burden.

Caregivers of children with ASD are at risk of developing psychiatric disorders, including depression and anxiety.^53^ Research done by Lai et al.^31^ compared parents of children with ASD to those of neurotypically developed children and found that parents of children with ASD experienced remarkably more difficulties regarding psychological well-being and coping. From this research, it is also evident that the personal well-being of caregivers suffered because of the role they were fulfilling. Caregivers reported feeling stressed about caring for the child with ASD and meeting all their responsibilities at home and work.

Functioning as a caregiver can lead to feelings of isolation, specifically related to the stigmatisation of the child with ASD and can contribute to interpersonal relationship strain.^54^ Caregivers in this study population were less likely to report that their relationships and social lives suffered severely. However, more than one-third did experience these feelings.

There is a paucity of research on the privacy of caregivers and how it is affected by the role they are fulfilling. However, despite the lack of research on this subject, the current study found that caregivers did not perceive their privacy to be impacted by their caregiving role.

The previous literature identified that physical health complaints and symptoms of fatigue were prominent features in caregivers.^28,55^ These symptoms were more pronounced when associated with depressive symptoms.^28,55^ In this study, caregivers did not feel that their physical health had been affected. A possible explanation could be that caregivers underreported these symptoms.

Most caregivers in this study felt that they still had enough time for themselves, had not lost control over their own lives and that they did not feel strained when they were around the child with ASD. This contrasts with a study by Ten Hoopen et al.^28^ which identified that more than half of the participating caregivers had difficulties combining their caregiving role with daily activities.

Behavioural problems in children with ASD can lead to anger and frustration.^56^ However, an interesting finding in this study was that most caregivers did not report feelings of anger but instead reported feeling that they could do more for the child with ASD.

Previous research reported that caregivers felt guilty for not spending all their free time with the child diagnosed with ASD and that they were not doing enough for them.^54^ This study agreed with previous publications and confirmed that caregivers felt uncertain about the child with ASD and, at the same time, reported that they could do a better job of taking care of the child.

### Burden prevalence characteristics

Caregiver burden is frequently reported in the literature.

Consistent with prior research regarding caregiver burden in the context of ASD, most caregivers in this study encountered levels of burden ranging from mild to moderate.^28,57^ An intriguing discovery from the study was that a significant portion of both male and female participants indicated experiencing mild to moderate levels of burden. Notably, there exists a lack of research directly comparing the caregiving burden between men and women who care for children with ASD.

Previous research has identified possible protective factors against high levels of burden. Pandey et al.^39^ reported that higher levels of education were associated with lower levels of burden, and Mumtaz et al.^58^ further identified that non-working caregivers scored higher on the ZBI questionnaire. Unemployed caregivers could be subjected to social isolation and experience economic burdens because of the loss of income.^59^ Possible protective factors against high burden levels in this study population could have been that most participants were educated, employed and received an income.

An additional protective factor could have been that most participants were religious. However, the full spectrum of the influence of religion on caregiver burden in South Africa still needs to be explored in future research.

## Limitations

To the author’s knowledge, this is one of the few studies conducted in South Africa on the caregiver burden in caregivers of children with ASD. Although it contributes to the understanding of the caregiver burden, several limitations must be acknowledged:

- This cross-sectional descriptive study and correlations between variables could not be drawn because of the limited sample size. Further research harnessing a larger sample size would be beneficial.
- The study’s cross-sectional nature precludes understanding the temporal sequence of factors that increase the caregiver burden.
- The study does not show any causal relationship between the demographic characteristics and the extent of the caregiver burden. Therefore risk and protective factors among the demographics could not be established with certainty.
- The study looked only at caregivers of children attending CMJAH and excluded caregivers of children with ASD attending community clinics, NGOs and the private sector.
- The study did not look at community (non-clinical) samples of caregivers. Therefore the results cannot be applied to all caregivers of children with ASD.
- The study did not measure the impact of the level of function and severity of symptoms in children with ASD on the caregiver burden.
- In cases where translation was used, the accuracy of information collected could only be partially ascertained.

## Conclusion

Autism spectrum disorder is a complex neurodevelopmental condition with many comorbidities. Taking care of a child with this disorder has a significant impact on the caregiver’s life. This is one of the few studies done in South Africa on caregiver burden in caregivers of children with ASD.

In this study, most of the caregivers were single mothers of the Christian faith. Most of the caregivers were educated and had part-time or full-time jobs. Despite most of the caregivers reporting that looking after a child with ASD was stressful, the minority reported that their social life and personal relationships were negatively affected.

Most of the caregivers did not report fatigue and ill physical health. Despite the difficult task of looking after a child with ASD, most caregivers did not report insufficient time for themselves. Caregivers in this study did not experience anger towards the child with ASD. Most experienced mild to moderate burden and felt guilty for not spending enough time with the child in their care.

Identifiable protective factors could be education, employment and religion, but more studies are needed to confirm the above findings.

### Recommendations

Despite its limitations, this study suggests clinical implications for assessing the caregivers’ burden among caregivers of children with ASD.

Improved knowledge about the caregiver burden might inspire the development of more culturally sensitive screening tools for the South African population. In addition, the awareness that caregivers experience stress and guilt might encourage the development of support groups in tertiary and community settings.

Developing programmes aimed at improving caregivers’ understanding of their burden, risk factors and protective factors can lead to a better quality of life for them. Additionally, educating the community and other healthcare providers about the challenges faced by caregivers of children with ASD can increase awareness and support for this population.

And finally, future research is needed to assess the prevalence of mental health disorders among caregivers, given the significant stress and burden they experience.
